# West Nile Virus Isolation from Equines in Argentina, 2006

**DOI:** 10.3201/eid1210.060852

**Published:** 2006-10

**Authors:** María Alejandra Morales, María Barrandeguy, Cintia Fabbri, Jorge B. Garcia, Aldana Vissani, Karina Trono, Gerónimo Gutierrez, Santiago Pigretti, Hernán Menchaca, Nelson Garrido, Nora Taylor, Fernando Fernandez, Silvana Levis, Delia Enría

**Affiliations:** * Instituto Nacional de Enfermedades Virales Humanas "Dr. Julio I. Maiztegui," Pergamino, Buenos Aires, Argentina;; † Instituto de Virología, CICVyA INTA, Castelar, Buenos Aires, Argentina;; ‡ San Antonio de Areco, Argentina;; § San Isidro, Argentina

**Keywords:** West Nile virus, Flavivirus, equines, encephalitis, isolation, RT-PCR, dispatch

## Abstract

West Nile virus (WNV) was isolated from the brains of 3 horses that died from encephalitis in February 2006. The horses were from different farms in central Argentina and had not traveled outside the country. This is the first isolation of WNV in South America.

Since West Nile virus (WNV) was detected in the Western Hemisphere in 1999 (*1*), the National Service of Animal Health (SENASA) has restricted the entry of WNV-susceptible species into the country, and the National Reference Center for Dengue and Arboviral Diagnosis of Argentina, Instituto Nacional de Enfermedades Virales Humanas (INEVH "Dr. Julio I. Maiztegui") incorporated new laboratory techniques, performed multidisciplinary training, and implemented laboratory WNV surveillance for birds, equines, and humans.

## The Study

**Figure 1:**
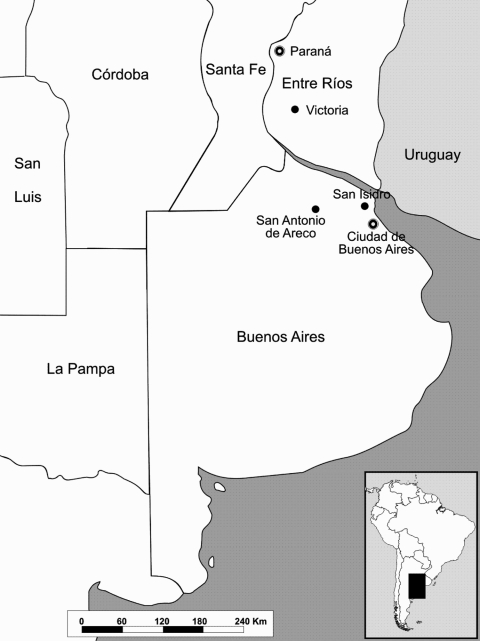
Locations of dead horses reported from February to March 2006 in central Argentina.

In late February 2006, two horses died after encephalitis developed at 2 stud farms near San Antonio de Areco, 110 km north of the city of Buenos Aires (Figure 1). Clinical signs included circling and acute onset ataxia, hypersensitivity to noise, hyperexcitability, and recumbency. Both mares (Eq001 and Eq002) died within 48 to 72 hours of onset of signs. Although each farm contained ≈300 horses, no other horses had clinical signs.

In March, another horse death was reported at the Jockey Club horse training center in San Isidro, province of Buenos Aires, within 48 hours after its arrival from a polo horse farm near Victoria (Entre Rios Province) (Figure 1). Clinical symptoms of the polo mare (Eq003) were weakness, paralysis, and recumbency.

Brain tissues from the 3 horses were extracted for virologic studies. Diagnostic tests for rabies virus (Dirección de Laboratorios y Control Técnico, SENASA) and for equine herpesvirus 1 (INTA Laboratory, Castelar, Argentina) were performed, with negative results. At INEVH, virus was isolated in African green monkey (Vero) cells, as described (*2*). Briefly, 20% homogenates of brain tissues were prepared and cultured in Vero cells for 14 days. Cultures were examined daily for evidence of viral cytopathic effect (CPE). Infected cells that showed CPEs were harvested and evaluated by immunofluorescent assay (IFA) (*3*) for flavivirus antigen, by using fluorescein isothiocyanate–labeled flavivirus polyclonal antisera (Centers for Disease Control and Prevention, Puerto Rico), and for WNV antigen by using the WNV-specific monoclonal immunoglobulin M (IgM) antibody H5-46 and IgG neutralizing monoclonal antibody 7H2. Cultures that did not show CPEs on the original isolation were blind passaged 1 more time onto fresh Vero monolayers. Three viral isolates were obtained. Two cultures (ArEq001 and ArEq003) showed CPEs on day 7 postinoculation of the original isolation; in the third culture (ArEq002), CPEs were observed only after the blind passage on day 7 after inoculation. WNV antigen was demonstrated when the 3 culture isolates were examined by IFA. Negative results were obtained for Saint Louis encephalitis virus by using the monoclonal antibody 6B5A-2. Negative results were also obtained for the alphaviruses Western equine encephalitis, Eastern equine encephalitis, and Venezuelan equine encephalitis by using mouse hyperimmune ascitic fluid. Titration of the supernatants by plaque assay gave titers of 10^5^ to 10^6^ PFU/μL.

For molecular identification of the virus isolates, viral RNA was extracted from 140 μL of infected Vero cell culture supernatant by using QIAamp viral RNA extraction kit (Qiagen, Inc., Valencia, CA, USA). Nested reverse transcription (nRT)– PCR assay was performed as described by Shi et al. (*4*). A DNA band of the correct size was visualized by gel electrophoreses of the RT-PCR product. Using the nRT-PCR previously described by Johnson et al. (*5*), the INTA laboratory also detected WNV RNA directly from brain tissue from the 3 necropsy specimens. In this case, total RNA was extracted from 50 to 100 mg of tissue by using Trizol reagent (Invitrogen Life Technologies, Carlsbad, CA, USA). WNV NY99 RNA lysate was employed as positive control. INEVH and INTA laboratories did not have infectious WNV in their virus collections.

**Figure 2:**
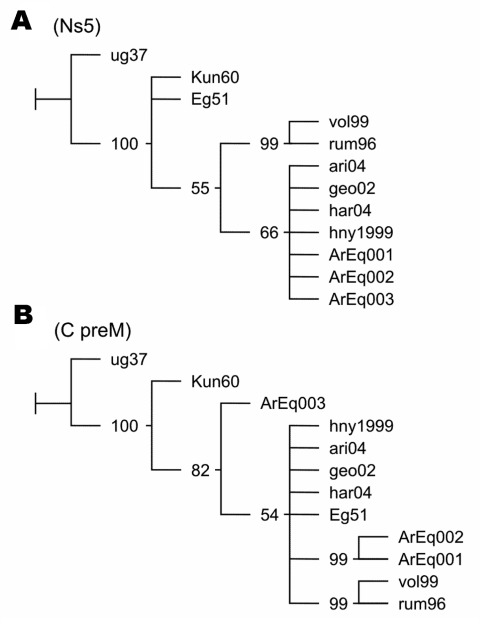
Phylogenetic trees of West Nile virus (WNV) nucleotide sequences. Maximum parsimony trees were obtained with TNT software (*6*). Values of jackknifing support are indicated at nodes. GenBank accession nos. ArEq001, ArEq002, and ArEq003: DQ537383, DQ537385, and DQ811782 (fragments NS5), DQ537382, DQ537384, and DQ811783 (fragments c/prM); ug37: M12294; vol99: AF317203, ari04: DQ164201; geo02: DQ164196; har04: DQ164206; hny1999: AF202541; Kun60: D00246; rum96: AF260969; Eg51:AF260968. A) NS5 fragment. B) C/preM fragment.

DNA fragments from C/preM and NS5 genes amplified by RT-PCR assays employing primers WN212/WN619c (5´-TTGTGTTGGCTCTCTTGGCGTTCTT-3´/5´-CAGCCGACAGCACTGGACATTCATA-3´) and WN9483/WN9794c (5´-CACCTACGCCCTAAACACTTTCACC-3´/5´-GGAACCTGCTGCCAATCATACCATC-3´) were sequenced. DNA products were analyzed on a 2% agarose gel, and the bands of the correct predicted size were excised and purified with the Gene Clean Kit (BIO 101, Carlsbad, CA, USA). The nucleotide sequences were determined by automatic dideoxy cycle sequencing techniques (Applied Biosystems, Foster City, CA, USA). Alignments were performed with BioEdit program by the Clustal Wallis method (available from http://www.mbio.ncsu.edu/BioEdit/bioedit.html). Phylogenetic maximum parsimony trees were generated with the TNT program (*6*). Jackknife resampling with 1,000 replicates was performed to evaluate the obtained trees. Phylogenetic analysis of NS5 fragments placed the Argentinean sequences in the North American cluster of lineage IA (Figure 2A). Sequences ArEq001 and ArEq002 showed a 100% nucleotide (nt) identity with hny1999 and differed by only 1 nt from sequence ArEq003. Phylogenetic analysis of the C/preM fragments also placed the Argentinean sequences in lineage IA (Figure 2B). Once again, ArEq001 was identical to ArEq002 but showed 9 nt differences from ArEq003. ArEq001 and ArEq002 grouped together because of 3 point mutations at nt 172, 208, and 245 in our fragments (corresponding to positions 390, 426, and 463 of WNV hny1999 strain. Whereas the first 2 mutations are silent, at position 463 the substitution of G for A results in a change of valine to isoleucine. Correct placement of ArEq003 is not clear because of several nucleotide changes.

## Conclusions

Since WNV was first detected in New York in the summer of 1999, it spread from the Atlantic Coast to the Pacific Coast in the United States and has affected Canada, Mexico, and some locations in the Caribbean basin, Central America, and northern Colombia (*7**,**8*). Our results are further evidence of the expanding geographic distribution of WNV.

WNV was isolated and detected by molecular techniques in Argentina from 3 horses that had no record of traveling outside the country during the preceding 4 months and that had not received vaccines against WNV. San Antonio de Areco may provide ideal conditions for detecting an enzootic cycle, given that >9,000 horses are raised there. Horses are probably "dead-end" or incidental hosts in the WNV transmission cycle; 10%–20% of the infections result in clinical disease, and the mortality rate in equines varies from 28% to 45% (*9*). Avian deaths were not observed in the affected places. Victoria, a potential site of infection for 1 of the studied equines, is 100 km southeast of Paraná City, where human flavivirus encephalitis cases have been diagnosed since February 2006. Cross-neutralization studies with a panel of flaviviruses are being conducted to identify the specific etiologic agent.

Analysis of the genomic sequences places the 3 Argentinean isolates in WNV lineage IA. Phylogenetic analysis of the NS5 fragment place them in the North American cluster, although an additional resolution of this cluster was not achieved because of high conservation levels and shortage of parsimony informative sites. A clear resolution of lineage IA clades was not obtained from the nucleotide sequences of the C/preM fragments. Nevertheless, a different location for Argentinean sequences was observed according to geographic origin. The substitution of valine to isoleucine, detected in sequences from San Antonio de Areco, was not observed in any sequence available from GenBank database. Additional investigation is necessary to establish possible implications for this change in terms of virulence and tropism of WNV.

WNV could be detected in southern South America because of the great economic value of the horses, which are subjected to continual veterinary evaluations. Animals of less economic value might have become infected and died without being noticed. The results reported here emphasize the need for an integrated surveillance system for WNV in Argentina with greater diagnostic capacity and the need for development of control strategies.
